# Combined Liver Stiffness and Α-fetoprotein Further beyond the Sustained Virologic Response Visit as Predictors of Long-Term Liver-Related Events in Patients with Chronic Hepatitis C

**DOI:** 10.1155/2022/5201443

**Published:** 2022-07-04

**Authors:** Sheng-Hung Chen, Hsueh-Chou Lai, Wen-Pang Su, Jung-Ta Kao, Po-Heng Chuang, Wei-Fan Hsu, Hung-Wei Wang, Tsung-Lin Hsieh, Hung-Yao Chen, Cheng-Yuan Peng

**Affiliations:** ^1^Department of Medicine, China Medical University, Taichung, Taiwan; ^2^Center for Digestive Medicine, Department of Internal Medicine, China Medical University Hospital, Taichung, Taiwan; ^3^Department of Chinese Medicine, China Medical University, Taichung, Taiwan

## Abstract

**Aims:**

Long-term risk stratification using combined liver stiffness (LS) and clinically relevant blood tests acquired at the baseline further beyond the sustained virologic response (SVR) visit for chronic hepatitis C (CHC) has not been thoroughly investigated. This study retrospectively investigated the prognostics of liver-related events (LREs) further beyond the SVR visit.

**Methods:**

Cox regression and random forest models identified the key factors, including longitudinal LS and noninvasive test results, that could predict LREs, including hepatocellular carcinoma, during prespecified follow-ups from 2010 to 2021. Kaplan–Meier survival analysis estimated the significance of between-group risk stratification.

**Results:**

Of the entire eligible cohort (*n* = 520) of CHC patients with SVR to antiviral therapy, 28 (5.4%) patients developed post-SVR LREs over a median follow-up period of 6.1 years (interquartile range = 3.5–8.7). The multivariate Cox regression analysis identified two significant predictors of LREs after the year 3 post-SVR (Y3PSVR) baseline (LRE, *n* = 15 of 28, 53.6%, median follow-up = 4.1 [1.6–6.4] years after Y3PSVR): LS at Y3PSVR (adjusted hazard ratio [aHR] = 3.980, 95% confidence interval [CI] = 2.085–7.597, *P* < 0.001), and *α*-fetoprotein (AFP) at Y3PSVR (aHR = 1.017, 95% CI = 1.001–1.034, *P*=0.034). LS ≥1.45 m/s and AFP ≥3.00 ng/mL for Y3PSVR yielded positive likelihood ratios of 4.24 and 2.62, respectively. Kaplan–Meier analysis revealed that among the stratified subgroups, the subgroup with concurrent LS ≥1.45 m/s and AFP ≥3.00 ng/mL at Y3PSVR exhibited the highest risk of LREs after Y3PSVR (log-rank *P* < 0.001).

**Conclusion:**

We recommend the combined use of concurrent LS and AFP in future prediction models for LREs in CHC. Patients with concurrently high LS and AFP values further beyond the SVR visit may require a recall policy involving intense surveillance.

## 1. Introduction

Global hepatitis C eradication is a major healthcare challenge. Viral eradication based on interferon (IFN) and direct-acting antiviral (DAA) agents benefits patient prognosis by reducing the incidence of extrahepatic morbidity [1], hepatocellular carcinoma (HCC) [2, 3], and liver-related events (LREs) [4]. However, HCC and hepatic decompensation can occur after viral eradication, especially in patients with post-treatment cirrhosis or advanced chronic liver disease [3, 5]. Therefore, posteradication surveillance is essential for patients with chronic hepatitis C (CHC) [6].

The incidence of HCC and LREs after sustained virologic response (SVR) has been widely investigated. Current surveillance programs typically rely on the use of liver elastography [7] and noninvasive tests (NITs) of blood-based markers [8] as baseline predictors. The risks of HCC and LREs can be stratified by values and decline in NITs from antiviral treatment baseline to on-treatment or off-treatment follow-ups [3, 5, 8–10]. Moreover, the liver stiffness (LS) value indicated by ultrasound measurement is a promising biomarker for risk stratification for antiviral treatment from baseline [11], after SVR [12–14], and before HCC treatment [15, 16]. Studies have revealed that the declining kinetics of LS values during treatment reflect the early resolution of hepatic necroinflammation and late, sustained regression of post-treatment fibrosis burden [17]. Post-SVR LS values, which may mitigate the confounding effects of hepatic necroinflammation, have been recommended for use as a baseline for post-SVR follow-up [18, 19].

The existing liver fibrosis burden may contribute to the common and pivotal upstream pathways that drive both hepatic carcinogenesis and decompensation [20–22]. However, the remaining post-treatment fibrosis burden, which can contribute to morbidity and mortality, typically declines over time [23, 24]. The optimal threshold values of the predictors acquired further beyond the SVR visit for risk stratification and recall policies [25] remain unclear.

Long-term risk stratification using combined LS and clinically recommended, acceptable, and available blood tests acquired at the baseline further beyond the SVR visit has not been thoroughly investigated. Therefore, this study used prespecified protocols to obtain LS values and NIT results further beyond the SVR visit to retrospectively investigate the utility of longitudinal measurements of precancerous LS and NITs in the prognostics of post-SVR LREs from two baselines (SVR and year 3 post-SVR [Y3PSVR]).

## 2. Materials and Methods

### 2.1. Participants

Patients with hepatitis C who had achieved an SVR to IFN- or DAA-based therapy and had completed at least two visits for LS measurements at China Medical University Hospital from August 2011 to November 2021 were retrospectively screened. SVR was defined as the absence of a detectable hepatitis C virus (HCV) RNA load on blood test results from 6 months or 3 months after the end of treatment (EOT) with IFN- or DAA-based therapy, respectively. Written informed consent was obtained from all participants. The study protocol, including the use of serial elastography, was approved by the Research Ethics Committee of the medical center (CMUH107-REC3-01). The protocol followed the principles of the Declaration of Helsinki of 1975.

HCV infection was detected using serum HCV RNA (detection limit: 15 IU/mL; COBAS Ampliprep/COBAS TaqMan HCV test, Roche Diagnostics, Branchburg, NJ, USA).

The patient exclusion criteria at screening were age <20 years, HCC, coinfection with hepatitis B virus or human immunodeficiency virus, alcohol dependence (score ≥2 on the CAGE questionnaire) [26], and a serum creatinine level of >2.5 mg/dL.

### 2.2. Blood Tests

The regular on- and off-treatment blood tests, including the serological *α*-fetoprotein (AFP) test and the tests used for calculating the aspartate aminotransferase (AST)-to-platelet ratio index (APRI) and the Fibrosis-4 index (FIB-4) [17] as concurrent noninvasive liver fibrosis indices, were performed in the central laboratory of the hospital.

After EOT, each patient underwent scheduled blood tests, including the AFP test, every 3 to 6 months.

### 2.3. Clinical Cirrhosis

Cirrhosis was clinically diagnosed at treatment baseline (TW0) according to the following criteria: a score of ≥8 in parenchymal liver disease on the 2D *B*-mode ultrasound [27], supplemented by the presence of ascites or gastroesophageal varices, or the stage of METAVIR F4 in liver histology [24], if available.

### 2.4. LS Measurements

The patients underwent scheduled prespecified LS measurements after 4 h of fasting at TW0, EOT, 12 weeks and 24 weeks after EOT, and every 6 to 12 months thereafter.

Point shear wave elastography is integrated into an ultrasound system (Acuson S2000 with a Siemens 4C1 curvilinear array, 2.67 MHz for push pulses, and 3.08 MHz for detection pulses; Siemens Healthineers, Erlangen, Germany) to measure the patients' LS.

One hepatologist (S. H. C.), who had 10 years of experience in LS measurement using point shear-wave elastography and was blinded to the patient data, performed all LS measurements through the intercostal approach. The measurement results (in meters per second [m/s] for the shear-wave velocities) were deemed reliable when the interquartile range (IQR) was <0.15 of the median of 10 successful LS measurements. The successful LS measurement rate was >60%. All other results were deemed unreliable and were excluded [28].

### 2.5. Endpoint Definitions

The LRE of interest was defined as the first de novo occurrence of any LRE. The LREs considered in this study were HCC and liver decompensation after the SVR visit (post-SVR) and after year 3 post-SVR (post-Y3PSVR). The liver decompensation events recorded were portal hypertensive gastrointestinal bleeding (diagnosed by endoscopy), portal hypertension-related ascites (diagnosed through paracentesis or imaging), spontaneous bacterial peritonitis (diagnosed through paracentesis), hepatic encephalopathy (diagnosed clinically), hepatorenal syndrome (diagnosed clinically), acute-on-chronic liver failure necessitating transplantation (diagnosed clinically), and liver-related mortality (diagnosed clinically). HCC was diagnosed according to the criteria of the American Association for the Study of Liver Diseases [29]. HCC was diagnosed following pathologic confirmation or by at least one imaging technique (contrast-enhanced computed tomography, contrast-enhanced magnetic resonance imaging, or contrast-enhanced ultrasound) for liver nodules at least 1 cm in diameter in patients with cirrhosis. The data of the patients who dropped out during the follow-up period were censored at the time they were lost to follow-up.

### 2.6. Statistical Analysis

The data are presented as medians (IQRs) or numbers (percentages). Between-group and overall differences were estimated using the Mann–Whitney U and Kruskal–Wallis tests for continuous variables and the chi-square or Fisher's exact test for categorical variables between unpaired or independent groups. Paired parameters from baseline to follow-up visits were compared using the Wilcoxon signed-rank test for continuous variables.

Cox regression models were used to identify the significant predictors of specific LREs after the SVR baseline in the entire cohort and to estimate these factors after the Y3PSVR baseline in the post-Y3PSVR subcohort. The participants with missing data were excluded from the modeling. Case numbers are disclosed for each model. Collinearity between the covariates was evaluated using a variance inflation factor (VIF).

A single decision tree classifier and an ensemble random forest model [30] were preliminarily trained with no validation to assist in predictor identification. Machine learning (ML) used the significant predictors identified using the Cox regression model to predict the binary occurrence (yes or no) of LREs, thereby minimizing the information loss. The performance of the trained model was evaluated using the area under the receiver operating characteristic (ROC) curve (AUC), accuracy ([true positives + true negatives]/total predictions), precision (true positives/[true positives + false positives]), recall (true positives/[true positives + false negatives]), and F1 (2/{[1/precision] + [1/recall]}). Instances with missing data were excluded from the classification.

To evaluate the clinical utility of the significant predictors in the prediction of LREs, a non-time-dependent ROC analysis was conducted to estimate the discriminative performance of each individual predictor. The AUC, sensitivity, specificity, predictive values, and likelihood ratios based on the cutoff values that maximized the Youden index were recorded. The significance levels of the differences in AUCs were evaluated using the DeLong test. Kaplan–Meier survival analysis was conducted to estimate the significance of between-group risk stratification using predictors classified according to the cutoffs. The key predictors were grouped into combination classes through the Kaplan–Meier analysis to enhance the predictive performance and utility of the model. The significance levels were compared using a log-rank test.

The data were analyzed using SPSS version 22.0 for Windows (IBM, Armonk, NY, USA). A two-sided *P* value <0.05 indicated statistical significance.

## 3. Results

### 3.1. Participant Characteristics

**Figure 1:**
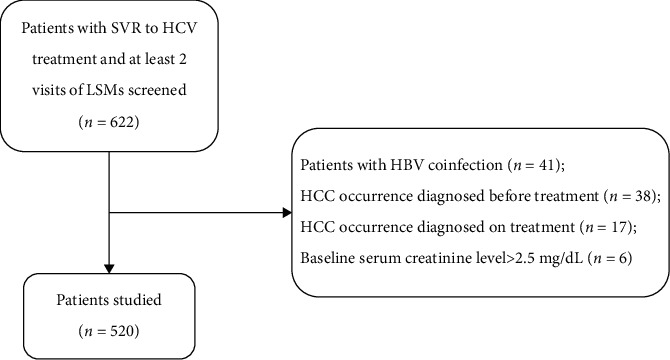
Patient recruitment flow chart. HBV, hepatitis B virus; HCC, hepatocellular carcinoma; HCV, hepatitis C virus; LSM, liver stiffness measurement; SVR, sustained virologic response.

Each of the 520 eligible participants with an SVR to HCV monoinfection underwent at least one session of LS measurements (Figure 1). The median age was 56 years (IQR, 48–63), and 260 (50%) of the participants were male.

Age, sex, baseline HCV RNA load, HCV genotypes, clinical cirrhosis (TW0), AST (TW0), AFP (TW0), AFP decline (from the SVR visit to Y3 post-SVR), estimated glomerular filtration rate (TW0), platelets (TW0), international normalized ratio (INR) (TW0), LS (TW0), LS (SVR), median post-SVR follow-up months, and median post-Y3PSVR follow-up months differed significantly between the group that underwent IFN-based therapy (*n* = 406) and the group that underwent DAA-based therapy (*n* = 114, Table 1).

The prevalence of clinical cirrhosis (TW0) was significantly higher (*P* < 0.001) in the DAA group (27.2%, 31/114) than in the IFN group (7.9%, 32/406) (Table 1). In the cirrhosis group, cirrhosis was histologically confirmed in 32 of 63 patients (50.8%). Baseline age, AST level, APRI, FIB-4, and LS were significantly higher and platelet counts were significantly lower in the group with baseline clinical cirrhosis (*n* = 63) than in the group without baseline cirrhosis (*n* = 457) (all *P* < 0.001; Table S1).

### 3.2. Longitudinal Kinetics of LS and NIT Values

The LS values (from 1.25 [1.08–1.61] to 1.15 [1.02–1.36], *P* < 0.001) and AFP values (from 2.99 [2.13–4.42] to 2.88 [2.13–3.94], *P*=0.005) decreased significantly from the SVR visit to Y3PSVR, with median percent decreases of 5.77% (−3.48% to 19.22%) and 4.04% (−15.61% to 20.33%), respectively.

The positive correlations and collinearity between LS (TW0) and APRI (TW0), LS (TW0) and FIB-4 (TW0), LS (SVR) and APRI (SVR), LS (SVR) and FIB-4 (SVR), LS (TW0) and LS (SVR), and LS (SVR) and LS (Y3PSVR) were all significant (all VIFs = 1.000). LS (Y3PSVR) and LS decline (SVR to Y3PSVR) exhibited significant negative correlations (VIF = 1.000).

### 3.3. Post-SVR LREs

Of the 520 included patients, 28 (5.4%) developed LREs after the SVR baseline. The relevant LRE was defined as the first de novo LRE to occur. The total counts of post-SVR LREs are as follows: HCC (*n* = 20), portal hypertensive gastrointestinal bleeding (*n* = 5), portal hypertension-related ascites (*n* = 4), hepatic encephalopathy (*n* = 2), spontaneous bacterial peritonitis (*n* = 0), hepatorenal syndrome (*n* = 0), acute-on-chronic liver failure necessitating transplantation (*n* = 2), and liver-related mortality (*n* = 5). The median follow-up duration for the entire cohort (*n* = 520) was 6.1 years (IQR = 3.5–8.7; 3128 person-years), and the incidence rates of LREs were 1.2%, 5.1%, and 5.8% at 3, 5, and 7 years, respectively.

### 3.4. Post-Y3PSVR LREs

Of the 28 patients who developed post-SVR LREs, 15 (53.6%) developed the LREs after Y3PSVR. The total counts of post-Y3PSVR LREs are as follows: HCC (*n* = 10), portal hypertensive gastrointestinal bleeding (*n* = 3), portal hypertension-related ascites (*n* = 3), hepatic encephalopathy (*n* = 1), spontaneous bacterial peritonitis (*n* = 0), hepatorenal syndrome (*n* = 0), acute-on-chronic liver failure necessitating transplantation (*n* = 0), and liver-related mortality (*n* = 2).

### 3.5. LRE Prediction Post-SVR

**Figure 2:**
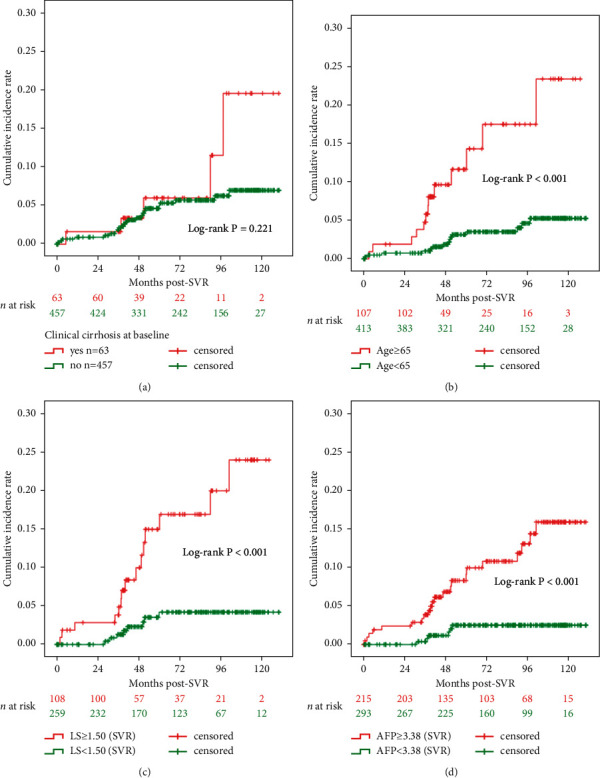
Kaplan–Meier survival analysis to predict LREs since the baseline of SVR visit. (a) LREs (*n* = 28), stratified by clinical cirrhosis (TW0), (b) LREs (*n* = 28), stratified by age (year) (TW0), (c) LREs (*n* = 23), stratified by LS (m/s) (SVR), and (d) LREs (*n* = 28), stratified by AFP (ng/mL) (SVR). Participants with missing data of LS or AFP were excluded from the analysis. AFP, *α*-fetoprotein; LRE, liver-related event; LS, liver stiffness; SVR, sustained virologic response (visit); TW0, treatment baseline.

After adjustment for baseline patient parameters acquired at the SVR visit, the multivariate Cox regression analysis identified the following three significant predictors of post-SVR LREs (*n* = 23, 5 LREs with missing covariates): age at TW0 (≥65 vs. <65 years; adjusted hazard ratio [aHR] = 2.882, 95% confidence interval [CI] = 1.187–6.996, *P*=0.019), LS at SVR (aHR = 2.398, 95% CI = 1.350–4.260, *P*=0.003), and AFP at SVR (aHR = 1.026, 95% CI = 1.000–1.052, *P*=0.039; Table 2). The median post-SVR follow-up period was 6.1 years. The results of Kaplan–Meier survival analyses of clinical cirrhosis (TW0), age (TW0), LS (SVR), and AFP (SVR) are presented in Figures 2(a)–2(d), respectively. After SVR, 5 LREs (7.9%) developed in patients with cirrhosis (TW0) (*n* = 63) and 23 (5.0%) in those without cirrhosis (TW0) (*n* = 457). Clinical cirrhosis at baseline did not significantly affect the stratification of the risk prediction for post-SVR LREs (log-rank *P*=0.221) (Figure 2(a)).

The single decision tree classifier model used LS (SVR), age, AFP (TW0), and AFP (SVR) to preliminarily classify the occurrence (yes or no) of LREs for each patient (Figure S1). The average performance indices of the trained decision tree model in the prediction of LREs were as follows: AUC = 0.790, accuracy = 0.967, *F*1 = 0.960, precision = 0.968, and recall = 0.967. The performance indices of the trained random forest model in the prediction of LREs were as follows: AUC = 0.911, accuracy = 0.952, *F*1 = 0.933, precision = 0.954, and recall = 0.952 (Table 3, Figure S2).

### 3.6. LRE Prediction Post-Y3PSVR

After adjustment for patient parameters acquired at the SVR visit, the multivariate Cox regression analysis identified two significant predictors of LREs after Y3PSVR (*n* = 12; 3 LRE with missing covariates): LS at Y3PSVR (aHR = 3.980, 95% CI = 2.085–7.597, *P* < 0.001) and AFP at Y3PSVR (aHR = 1.017, 95% CI = 1.001–1.034, *P*=0.034; Table 4). The median post-Y3PSVR follow-up period was 4.1 years (IQR = 1.6–6.4; 1804 person-years starting from the Y3PSVR baseline).

LS ≥1.45 m/s and AFP ≥3.00 ng/mL for Y3PSVR yielded AUCs of 0.824 and 0.664, respectively, and positive likelihood ratios of 4.24 and 2.63, respectively, in the prediction of post-Y3PSVR LREs (Table 5).

**Figure 3:**
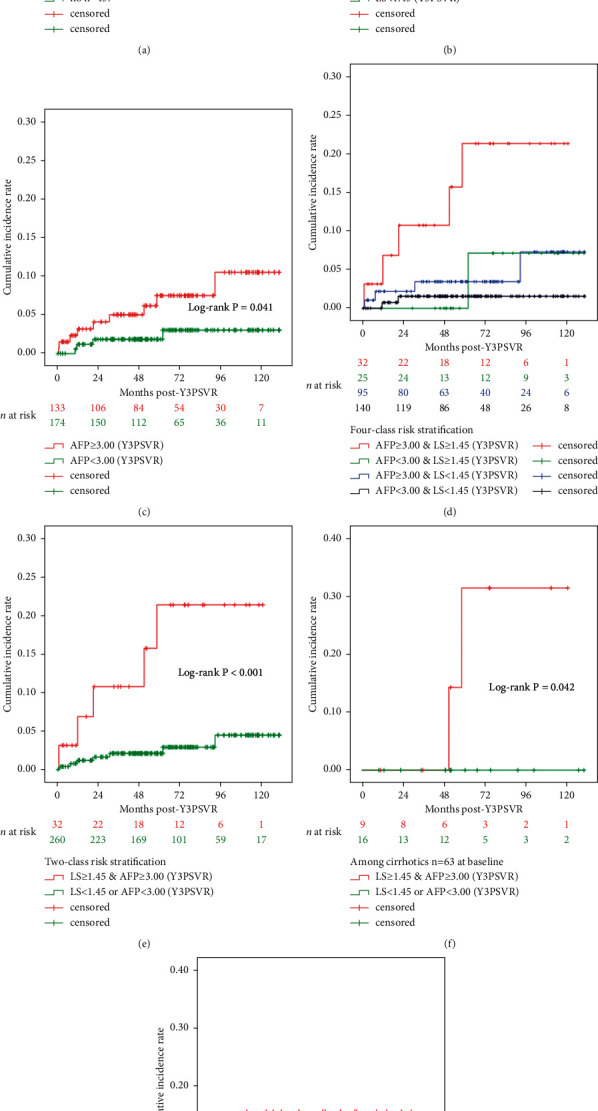
Kaplan–Meier survival analysis for predicting LREs from the baseline of Y3 post-SVR. (a) LREs (*n* = 15), stratified by clinical cirrhosis (TW0); (b) LREs (*n* = 14), stratified by LS (Y3PSVR); (c) LREs (*n* = 13, stratified by AFP (Y3PSVR); (d) LREs (*n* = 12), stratified by concurrent LS and AFP (Y3PSVR), pairwise log-rank *P*'s were significant only in classes “AFP ≥3.00 and LS ≥1.45” versus “AFP <3.00 and LS <1.45” (*P* < 0.001), and “AFP ≥3.00 and LS ≥1.45” versus “AFP ≥3.00 and LS <1.45” (*P*=0.020); (e) LREs (*n* = 12), stratified by concurrent LS and AFP (Y3PSVR); (f) LREs (*n* = 2), stratified and validated among patients with cirrhosis (*n* = 63, at baseline); (g) LREs (*n* = 13), stratified and validated among patients without cirrhosis (*n* = 457, at baseline). Participants with missing data of LS (Y3PSVR) or AFP (Y3PSVR) were excluded from the analysis. AFP, *α*-fetoprotein (ng/mL); LRE, liver-related event; LS, liver stiffness (m/s); SVR, sustained virologic response (visit); TW0, treatment baseline; Y3PSVR, year 3 post-sustained virologic response.

The data of 118 patients were censored before the first event after Y3PSVR. Similarly, after Y3PSVR, 3 LREs (7.0%) developed in patients with cirrhosis (*n* = 43) and 12 (3.3%) in those without cirrhosis (*n* = 359). Clinical cirrhosis at baseline did not significantly affect the stratification of risk prediction for post-Y3PSVR LREs (log-rank *P*=0.144, Kaplan–Meier analysis) (Figure 3(a)).

The Kaplan–Meier analyses (Figure 3) identified two classes: high-risk and low-risk (5/32 and 7/260 LREs, respectively) based on Y3PSVR LS and AFP values (log-rank *P* < 0.001) to optimize the stratification of the post-Y3PSVR risk of LREs. The high-risk subgroup (LS ≥1.45 m/s and AFP ≥3.00 ng/mL at Y3PSVR) exhibited a higher risk of LREs after Y3PSVR. The proportion of patients with LREs after Y3PSVR was 15.6% (5/32), with an incidence rate of 3.4 per 100 person-years and a 5-year cumulative incidence rate of 21.3%. By contrast, the low-risk subgroup (AFP <3.00 ng/mL or LS <1.45 m/s at Y3PSVR) exhibited a lower risk of LREs after Y3PSVR. The proportion of patients with LREs post-Y3PSVR was 2.7% (7/260), with an incidence rate of 0.5 per 100 person-years and a 5-year cumulative incidence rate of 2.1%.

While validating the Y3PSVR thresholds of LS and AFP, the results of Kaplan–Meier analyses were both significant in the group with cirrhosis at baseline (*n* = 63) (*P*=0.042, Figure 3(f)) and in that without cirrhosis at the baseline (*n* = 457) (*P*=0.006, Figure 3(g)), respectively.

## 4. Discussion

Surveillance using prediction factors over time may assist in identifying growing post-treatment patient populations at high or minimal risk of LREs. Nevertheless, risk stratification according to the baseline values (measurement of noninvasive markers) further beyond the SVR visit for CHC has received insufficient attention. The optimal threshold values of the predictors acquired further beyond the SVR visit for risk stratification remain unclear. The present study identified LS and AFP as key predictors of post-SVR LREs (Table 2). The predictor values acquired at Y3PSVR were subsequently applied to evaluate the discriminative performance in the prediction of LREs further beyond the SVR visit (post-Y3PSVR) in the subcohort with available predictor values at the Y3PSVR baseline (Table 4).

The incidences of LREs after the SVR and Y3PSVR baselines were not zero (Table 1). The residual post-treatment liver fibrosis burden may contribute to the common and pivotal upstream mechanisms that drive the development of LREs related to hepatocarcinogenesis and portal hypertension. These LREs remain emerging problems in the growing population of patients achieving viral eradication. After viral eradication, HCC risk gene signatures [20] may not be fully reversed in patients who have developed HCC. Previous studies have also revealed that epigenetic alterations persist and predispose patients to hepatocarcinogenesis after SVR. These remaining epigenetic signatures, including HCV protein-associated post-translational modifications of histones, may constitute the treatment targets for post-SVR carcinogenic risk reduction [21].

Post-treatment LS values may reflect the burden of remnant liver fibrosis. Post-treatment LS values may change over time by mitigating the confounding effects of hepatic necroinflammation on LS measurements [31]. A study revealed that post-treatment AFP >5.4 ng/mL is a predictor of a lack of histological fibrosis regression [9]. However, these predictors, which serve as surrogate markers of liver fibrosis, exhibit changing and declining kinetics across the treatment timeline [9, 17, 24]. A study reported that, compared with pretreatment liver fibrosis, post-treatment liver fibrosis is a more accurate prognostic marker of LREs, including HCC [23]. In the present study, the clinical diagnosis of cirrhosis at treatment baseline was not sufficiently discriminatory for post-SVR or post-Y3PSVR risk stratification to predict LREs after treatment completion (Tables 2 and 4; Figures 2(a) and 3(a)). In other words, the absence of cirrhosis at baseline did not preclude the need for LRE surveillance. Overall, 23 (5.0%) and 12 (3.3%) patients without baseline cirrhosis developed LREs after SVR and Y3PSVR, respectively (Figures 2(a) and 3(a)). The significant declines in LS and AFP values from the SVR visit to Y3PSVR imply the importance of validating the utility of predictor values acquired at Y3PSVR as baseline values in post-Y3PSVR prognostics (Table 4; Figure 3). Furthermore, the AUC of LS (Y3PSVR) being numerically higher than that of LS (SVR) is consistent with the results of a previous study [23]. LS and AFP values obtained at Y3PSVR may be incorporated into models for predicting post-Y3PSVR LREs to improve the efficacy and cost-effectiveness of recall policies. For patients who did not complete early on- or off-treatment surveillance sessions through blood tests, histology, ultrasound, or LS measurements, the measurements of Y3PSVR may compensate for the missed surveillance, thereby enabling the continuation of long-term surveillance. Regardless of the availability of data from early on- and off-treatment surveillance sessions, concurrently high LS and AFP values indicating leftover liver fibrosis and regeneration burden at Y3PSVR alert both physicians and patients that continuing or starting careful long-term surveillance is necessary.

The thresholds of precancerous AFP in the present study were much lower than those for HCC detection in naïve or posteradication settings [32, 33]. AFP, an oncofetal glycoprotein discovered decades ago, has also been used as a serum and gene expression marker to evaluate hepatocyte regeneration activity closely related to concurrent hepatic necroinflammation or damage predisposing the liver to progression to advanced fibrosis, cirrhosis, or HCC [34]. Growing evidence has revealed that mildly elevated AFP levels (<150 ng/mL) might reflect the status of liver regeneration and damage [35, 36]. AFP may be used as an additional predictor of post-SVR LREs to enhance the performance of predictive models for liver-related pathogenesis, which may not be explained by liver fibrosis or its surrogate markers alone [3].

The present study has several limitations. Firstly, NIT results, including APRI and FIB-4, were not included in the proposed predictive models for LREs because they were already known to exhibit promising predictive performance [8], and they exhibited significant collinearity with concurrent LS values in the present study. The declines in LS from the SVR visit to Y3PSVR were also excluded. The utility of absolute values of biomarkers, such as LS and AFP, acquired at post-treatment visits instead of the delta (decline) values over time may facilitate the generalization of simple noninvasive surveillance programs, with no need for calculations in clinical settings, for the risk stratification of post-treatment LREs. In the Asia-Pacific region, guidelines [33] recommend the assessment of serum AFP levels on and off antiviral treatment every 6 months for the HCC surveillance of patients with chronic liver diseases. By contrast, regular and longitudinal measurements of post-SVR patient parameters, such as platelet count, have not been strongly recommended. Therefore, NIT results, such as platelet-based indices, had a high rate of missing data in the present cohort, in both the post-SVR cohort and post-Y3PSVR subcohort. Secondly, ML models may exhibit higher accuracy than do regression models [3]. Preliminary ML-based algorithms were, therefore, applied to assist in identifying the key predictors of LREs in the present study. However, the limited number of post-Y3PSVR LREs hampered the model training and optimization. Thirdly, the values for the same markers acquired at different visits (e.g., LS at SVR and LS at Y3PSVR) were not simultaneously included in the same Cox regression modeling because of the dependence and collinearity between the same markers. In addition, the values collected at the time of SVR were not used as the baseline for post-Y3PSVR surveillance. Fourth, baseline age, which was one of the significant covariates identified through Cox regression analysis for post-SVR LRE prediction (Table 2), did not retain significance for post-Y3PSVR LRE prediction (Table 4). Therefore, age was not included in the subsequent Kaplan–Meier analysis (Figure 3). Studies involving larger cohorts are warranted to reappraise the significance of age in prognostics further beyond the SVR visit. Fifth, longitudinal spleen stiffness values closely reflect changes in portal hypertension in patients with advanced chronic liver disease treated with DAA [37]. Clinically significant portal hypertension and related LREs may not be radically abolished, despite SVR or a decrease in LS [4, 37]. Future LRE-predicting algorithms should employ a combination [38] of LS and spleen stiffness measurements, including delta values [39, 40], to maximize the diagnostic performances, as well as by considering remnant portal hypertension after antiviral treatment completion. Sixth, concerns and controversies regarding differences in HCC risk after viral eradication by IFN- or DAA-based therapies have been clarified. Recent meta-analyses have revealed no significant differences [2]. Therefore, the present study combined both IFN- or DAA-based cohorts despite their distinct characteristics (Table 1). Seventh, censoring may lead to biased estimates during the follow-up period and potential overestimation of LREs after Y3PSVR. Future studies may also need to evaluate LRE recurrence to optimize risk estimation for longer terms after SVR. Eighth, the LREs may have been subclinical before the first clinical identification or diagnosis [41]. Ninth, clinical cirrhosis was only evaluated at treatment baseline rather than at the two points after treatment completion: SVR and Y3PSVR. Lastly, the utility of the identified predictors can benefit surveillance by facilitating the identification of patients at high risk for LREs further beyond the SVR visit, for whom recall policies should be implemented. Additional studies are warranted to establish a prespecified actionable recall policy involving additional and more intensive return visits and diagnostic tests and to measure its cost-effectiveness.

## 5. Conclusion

We recommend the combined utility of concurrent LS and AFP acquired further beyond the SVR visit in future models for predicting the risk of LREs further beyond the SVR visit in patients with CHC. Patients with concurrently high LS and AFP values should undergo prespecified lifelong surveillance after viral eradication. Future studies should employ a larger cohort to validate the results of the present study, develop a prespecified recall policy, and evaluate the cost-effectiveness of the policy.

## Figures and Tables

**Table 1:** Patient characteristics.

Variables	IFN-based therapy		DAA-based therapy		*P* value
	Total *n*	Median (IQR) or *n*	Total *n*	Median (IQR) or *n*	
Age, years (TW0)	406	54 (45–60)	114	65 (58–73)	<0.001
Sex, male/female	406	214/192	114	46/68	0.026
Body mass index, kg/m^2^ (TW0)	406	24.2 (22.3–26.3)	114	24.2 (22.8–26.0)	0.592
HCV RNA log_10_, IU/mL (TW0)	406	6.22 (5.42–6.71)	114	6.76 (6.09–7.08)	<0.001
HCV genotype, 1/non-1	406	203/203	114	93/21	<0.001
Clinical cirrhosis, % (TW0)	406	32/406 (7.9%)	114	31/114 (27.2%)	<0.001
AST, U/L (TW0)	406	60 (40–98)	111	56 (37–85)	0.136
ALT, U/L (TW0)	406	84 (52–137)	114	66 (41–106)	0.001
AFP, ng/mL (TW0)	406	4.71 (3.03–9.32)	104	5.51 (3.06–11.26)	0.181
AFP, ng/mL (SVR)	403	2.90 (2.06–4.15)	105	3.46 (2.46–5.56)	0.004
AFP, ng/mL (Y3PSVR)	315	2.84 (2.11–3.95)	108	3.19 (2.21–3.94)	0.334
AFP decline, % (from SVR to Y3PSVR)	314	0 (−17.23–16.99)	100	11.97 (−3.00–26.70)	<0.001
Albumin, g/dL (TW0)	401	4.30 (4.10–4.60)	109	4.30 (4.10–4.50)	0.468
Bilirubin, mg/dL (TW0)	406	0.93 (0.70–1.14)	113	0.90 (0.70–1.10)	0.235
eGFR, mL/min/1.73 m^2^ (TW0)	406	99.52 (70.07–144)	114	92.55 (76.14–106)	0.036
HbA1c, % (TW0)	405	5.7 (5.4–6.0)	99	5.7 (5.4–6.1)	0.900
WBC, ×10^3^/*μ*L (TW0)	406	5.30 (4.30–6.52)	114	4.85 (3.99–6.32)	0.059
Hemoglobin, g/dL (TW0)	406	14.0 (13.1–15.1)	114	14.1 (13.0–15.0)	0.830
Platelet, ×10^3^/*μ*L (TW0)	406	165 (125–204)	114	143 (107–185)	0.012
INR (TW0)	406	1.04 (0.99–1.10)	113	1.02 (0.99–1.09)	0.225
LS, m/s (TW0)	309	1.46 (1.21–2.06)	78	1.89 (1.28–2.27)	0.014
LS, m/s (SVR)	295	1.21 (1.06–1.48)	72	1.53 (1.25–2.06)	<0.001
Median follow-up, months (post-SVR)	406	88.8 (54.0–110.6)	114	41.7 (39.1–55.1)	<0.001
LREs (post-SVR)		24		4	NA
Incidence rate, /100 person-year		0.8		1.0	NA
HCC (post-SVR)		17		3	NA
LS, m/s (Y3PSVR)	370	1.15 (1.02–1.34)	86	1.21 (1.05–1.42)	0.118
LS decline, % (from SVR to Y3PSVR)	268	4.89 (−4.25–18.49)	54	8.94 (−1.23–22.02)	0.131
Median follow-up, months (post-Y3PSVR)	352	55.86 (32.75–80.16)	55	13.00 (5.00–17.00)	<0.001
LREs (post-Y3PSVR)		14		1	NA
HCC (post-Y3PSVR)		10		0	NA

Data are presented as medians (interquartile ranges) or *n*. Decline = (preceding minus next values)/preceding value. AFP, *α*-fetoprotein; ALT, alanine aminotransferase; AST, aspartate aminotransferase; DAA, direct-acting antiviral; eGFR, estimated glomerular filtration rate (modification of diet in renal disease formula); HbA1c, hemoglobin A1c; HCC, hepatocellular carcinoma; HCV, hepatitis C virus; IFN, interferon; INR, international normalized ratio; LRE, liver-related event; LS, liver stiffness; SVR, sustained virologic response (visit); TW0, treatment baseline; WBC, white blood cell; Y3PSVR, year 3 post-SVR.

**Table 2:** Cox regression analysis for prediction of LREs (*n* = 23, multivariate analysis) after a sustained virologic response.

Variables	*n*/*n*	Crude HR	(95%CI)	*P* value	Adjusted HR	(95% CI)	*P* value
Age, ≥65 vs. <65 years (TW0)	28/514	1.068	1.028–1.109	<0.001	2.882	1.187–6.996	0.019
Sex, male vs female	28/514	0.776	0.369–1.633	0.504			
Body mass index, kg/m^2^ (TW0)	28/514	1.076	0.972–1.191	0.159			
HCV RNA log_10_, IU/mL (TW0)	28/514	1.000	1.000–1.000	0.265			
Genotype, 1 vs non-1	28/514	1.245	0.583–2.660	0.572			
Clinical cirrhosis (TW0)	28/520	1.817	0.688–4.795	0.228			
AST, U/L (TW0)	28/511	1.006	1.002–1.010	0.005			
ALT, U/L (TW0)	28/514	1.003	0.999–1.006	0.168			
AFP, ng/mL (TW0)	28/504	0.999	0.992–1.006	0.851			
Albumin, g/dL (TW0)	28/504	0.316	0.136–0.736	0.008			
Bilirubin, mg/dL (TW0)	28/513	1.458	0.587–3.621	0.417			
eGFR, mL/min/1.73 m^2^ (TW0)	28/514	0.994	0.985–1.004	0.243			
HbA1c, % (TW0)	27/498	1.166	0.845–1.609	0.351			
WBCs, /*μ*L (TW0)	28/514	1.000	1.000–1.000	0.823			
Hemoglobin, g/dL (TW0)	28/514	0.768	0.596–0.989	0.041			
Platelets, ×10^3^/*μ*L (TW0)	28/514	0.989	0.982–0.997	0.004			
INR (TW0)	28/513	1.062	0.043–26.14	0.970			
AST, U/L (SVR)	28/512	1.019	1.011–1.028	<0.001			
ALT, U/L (SVR)	28/514	1.013	1.004–1.021	0.004	0.996	0.979–1.014	0.667
AFP, ng/mL (SVR)	28/502	1.048	1.030–1.067	<0.001	1.026	1.000–1.052	0.039
Albumin, g/dL (SVR)	28/505	0.333	0.130–0.854	0.022	0.685	0.251–1.872	0.461
Bilirubin, mg/dL (SVR)	28/510	1.382	0.541–3.529	0.498	1.881	0.694–5.100	0.214
eGFR, mL/min/1.73 m^2^ (SVR)	28/505	0.992	0.982–1.002	0.130			
HbA1c, % (SVR)	27/471	1.112	0.740–1.672	0.608			
WBC, /*μ*L (SVR)	28/511	1.000	1.000–1.000	0.076			
Hemoglobin, g/dL (SVR)	28/512	0.795	0.624–1.012	0.062			
Platelet, ×10^3^/*μ*L (SVR)	28/512	0.986	0.979–0.994	<0.001	0.996	0.987–1.004	0.311
INR (SVR)	28/487	12.222	1.037–144	0.047			
LS, m/s (TW0)	24/376	2.745	1.661–4.536	<0.001			
LS, m/s (SVR)	23/356	2.954	1.888–4.621	<0.001	2.398	1.350–4.260	0.003
APRI (TW0)	28/511	1.236	1.086–1.408	0.001			
APRI (SVR)	28/510	1.879	1.511–2.336	<0.001			
FIB-4 (TW0)	28/511	1.203	1.104–1.311	<0.001			
FIB-4 (SVR)	28/510	1.375	1.243–1.521	<0.001			

*n*/*n* indicates the number of events and the sample size (cases with missing values were excluded; six cases were censored before the first event after SVR; 350 cases entered the multivariate analysis). AFP, *α*-fetoprotein; ALT, alanine aminotransferase; APRI, aspartate-aminotransferase-to-platelet ratio index; AST, aspartate aminotransferase; CI, confidence interval; eGFR, estimated glomerular filtration rate (modification of diet in renal disease formula); FIB-4, fibrosis-4 index; HbA1c, hemoglobin A1c; HCC, hepatocellular carcinoma; HCV, hepatitis C virus; HR, hazard ratio; INR, international normalized ratio; LRE, liver-related event; LS, liver stiffness; SVR, sustained virologic response (visit); TW0, treatment baseline; WBC, white blood cell.

**Table 3:** Performance of decision tree and random forest classifiers in predicting LREs.

	AUC	Accuracy	F1	Precision	Recall
*Average*					
Decision tree	0.790	0.967	0.960	0.968	0.967
Random forest	0.911	0.952	0.933	0.954	0.952

*LRE: yes*					
Decision tree	0.790	0.967	0.564	1.000	0.393
Random forest	0.911	0.952	0.194	1.000	0.107

*LRE: no*					
Decision tree	0.790	0.967	0.983	0.967	1.000
Random forest	0.911	0.952	0.975	0.952	1.000

Performances of the trained models were evaluated using AUC, accuracy ([true positives + true negatives]/total predictions), precision (true positives/[true positives + false positives]), recall (true positives/[true positives + false negatives]), and F1 (2/[(1/precision) + (1/recall)]). AUC, area under the curve; LRE, liver-related events.

**Table 4:** Cox regression analysis for prediction of LREs (*n* = 12, multivariate analysis) after year 3 post-SVR.

Variables	*n*/*n*	Crude HR	(95% CI)	*P* value	Adjusted HR	(95% CI)	*P* value
Age, ≥65 vs. <65 years (TW0)	15/402	1.055	0.997–1.117	0.064	2.362	0.643–8.675	0.195
Sex, male vs female	15/402	0.563	0.200–1.583	0.276			
Body mass index, kg/m^2^ (TW0)	15/402	1.054	0.915–1.214	0.467			
HCV RNA log_10_, IU/mL (TW0)	15/402	1.000	1.000–1.000	0.430			
Genotype, 1 vs non-1	15/402	1.031	0.374–2.845	0.952			
Clinical cirrhosis (TW0)	15/402	2.494	0.702–8.862	0.158			
AST, U/L (TW0)	15/401	1.009	1.004–1.013	<0.001			
ALT, U/L (TW0)	15/402	1.005	1.000–1.009	0.029			
AFP, ng/mL (TW0)	15/395	0.997	0.979–1.015	0.729			
Albumin, g/dL (TW0)	15/396	0.327	0.098–1.096	0.070			
Bilirubin, mg/dL (TW0)	15/402	2.478	0.811–7.574	0.111			
eGFR, mL/min/1.73 m^2^ (TW0)	15/402	0.995	0.983–1.008	0.478			
HbA1c, % (TW0)	15/398	1.048	0.627–1.754	0.857			
WBCs, /*μ*L (TW0)	15/402	0.999	0.999–1.000	0.010			
Hemoglobin, g/dL (TW0)	15/402	0.780	0.550–1.105	0.163			
Platelets, ×10^3^/*μ*L (TW0)	15/402	0.983	0.972–0.995	0.004			
INR (TW0)	15/401	213.579	0.375–1200	0.098			
AST, U/L (SVR)	15/401	1.022	1.009–1.036	0.001			
ALT, U/L (SVR)	15/402	1.009	0.994–1.024	0.236			
AFP, ng/mL (SVR)	15/392	1.050	1.024–1.076	<0.001			
AFP, ng/mL (Y3PSVR)	13/306	1.021	1.011–1.030	<0.001	1.017	1.001–1.034	0.034
AFP decline, % (from SVR to Y3PSVR)	13/305	0.930	0.897–0.963	<0.001			
Albumin, g/dL (SVR)	15/394	1.482	0.279–7.880	0.645			
Bilirubin, mg/dL (SVR)	15/398	1.181	0.309–4.511	0.808			
eGFR, mL/min/1.73 m^2^ (SVR)	15/394	0.993	0.980–1.006	0.315			
HbA1c, % (SVR)	14/365	0.789	0.362–1.720	0.552			
WBC, /*μ*L (SVR)	15/399	1.000	0.999–1.000	0.081			
Hemoglobin, g/dL (SVR)	15/400	0.894	0.633–1.260	0.522			
Platelet, ×10^3^/*μ*L (SVR)	15/400	0.984	0.973–0.995	0.004	0.994	0.982–1.005	0.276
INR (SVR)	15/380	14.920	0.265–838	0.189			
LS, m/s (TW0)	13/290	2.153	1.093–4.241	0.027			
LS, m/s (SVR)	12/255	2.901	1.611–5.223	<0.001			
LS, m/s (Y3PSVR)	14/369	3.208	1.631–6.306	0.001	3.980	2.085–7.597	<0.001
LS decline, % (from SVR to Y3PSVR)	11/239	0.380	0.031–4.669	0.450			
APRI (TW0)	15/401	1.330	1.150–1.537	<0.001			
APRI (SVR)	15/399	2.016	1.466–2.773	<0.001			
FIB-4 (TW0)	15/401	1.303	1.162–1.462	<0.001			
FIB-4 (SVR)	15/399	1.544	1.319–1.806	<0.001			

*n*/*n* indicates the number of events and the sample size (cases with missing values were excluded; 118 cases were censored before the first event after Y3PSVR; 332 cases entered the multivariate analysis). Decline = (preceding value minus next values)/preceding value. AFP, *α*-fetoprotein; ALT, alanine aminotransferase; APRI, aspartate-aminotransferase-to-platelet ratio index; AST, aspartate aminotransferase; CI, confidence interval; eGFR, estimated glomerular filtration rate (Modification of Diet in Renal Disease formula); FIB-4, fibrosis-4 index; HbA1c, hemoglobin A1c; HCC, hepatocellular carcinoma; HCV, hepatitis C virus; HR, hazard ratio; INR, international normalized ratio; LRE, liver-related event; LS, liver stiffness; SVR, sustained virologic response (visit); TW0, treatment baseline; WBC, white blood cell; Y3PSVR, year 3 post-SVR.

**Table 5:** Receiver operating characteristic analysis for the prediction of LREs after SVR and Y3PSVR.

Variables	*n*/*n*	AUC	95% CI	*P* value	Cutoff	Se	Sp	PPV	NPV	+LR	−LR
*Predicting post-SVR LREs*											
Age (TW0)	28/520	0.652	0.547–0.757	0.007	65	46.4	80.9	12.2	96.4	2.43	0.66
LS (SVR)	23/367	0.775	0.678–0.871	<0.001	1.50	73.9	27.0	16.3	97.5	2.73	0.36
AFP (SVR)	28/508	0.724	0.633–0.815	<0.001	3.38	78.6	59.8	10.2	98.0	1.96	0.36

*Predicting post-Y3PSVR LREs*											
Age (TW0)	15/520	0.563	0.428–0.697	0.407	NA	NA	NA	NA	NA	NA	NA
LS (SVR)	10/367	0.774	0.629–0.919	0.003	1.98	70.0	85.7	12.1	90.0	4.90	0.35
LS (Y3PSVR)	14/374	0.824	0.704–0.945	<0.001	1.45	73.3	82.7	14.1	98.8	4.24	0.32
AFP (Y3PSVR)	13/361	0.664	0.499–0.828	0.038	3.00	64.3	75.5	8.9	98.3	2.62	0.47

Age, years; LS, m/s; AFP, ng/mL. *n*/*n* indicates the number of events and the sample size. Comparison of AUCs for LS (SVR) and LS (Y3PSVR) in predicting post-Y3PSVR LREs: *P*=0.535. AFP, *α*-fetoprotein; AUC, area under the curve; CI, confidence interval; −LR, negative likelihood ratio; +LR, positive likelihood ratio; LRE, liver-related events; LS, liver stiffness; NA, not available; NPV, negative predictive value; PPV, positive predictive value; Se, sensitivity; Sp, specificity; SVR, sustained virologic response (visit); TW0, treatment baseline; Y3PSVR, year 3 post-SVR.

## Data Availability

All data generated or analyzed during this study are included in this article. Further details are available from the corresponding author upon request.

## Abbreviations

AFP: Α-fetoprotein
APRI: Aspartate-aminotransferase-to-platelet ratio index
AUC: Area under the curve
CHC: Chronic hepatitis C
CI: Confidence interval
DAA: Direct-acting antiviral
EOT: End of treatment
FIB-4: Fibrosis-4 index
HCC: Hepatocellular carcinoma
HCV: Hepatitis C virus
HR: Hazard ratio
IFN: Interferon
IQR: Interquartile range
LRE: Liver-related events
−LR: Negative likelihood ratio
+LR: Positive likelihood ratio
LS: Liver stiffness
NIT: Noninvasive test
NPV: Negative predictive value
PPV: Positive predictive value
ROC: Receiver operating characteristics
SVR: Sustained virologic response
VIF: Variance inflation factor
Y3PSVR: Year 3 post-sustained virologic response.

## Supplementary Materials

Supplementary Materials Figure S1. Single decision tree classifier preliminarily trained to assist in predictor identification. Figure S2. ROC curves for LRE classification with single decision tree and ensemble random forest classifiers. Table S1. Comparison of baseline characteristics between patients without (*n* = 457) and with (*n* = 63) clinical cirrhosis.
