# Supplementary material for: Combined Liver Stiffness and Α-fetoprotein Further beyond the Sustained Virologic Response Visit as Predictors of Long-Term Liver-Related Events in Patients with Chronic Hepatitis C
Source: Can J Gastroenterol Hepatol. 2022 Jul 4;2022:5201443. doi: 10.1155/2022/5201443 (PMC9273470; doi:10.1155/2022/5201443)
Supplement: Supplementary Materials — Figure S1. Single decision tree classifier preliminarily trained to assist in predictor identification. Figure S2. ROC curves for LRE classification with single decision tree and ensemble random forest classifiers. Table S1. Comparison of baseline characteristics between patients without (n = 457) and with (n = 63) clinical cirrhosis. [file 5201443.f1.docx]

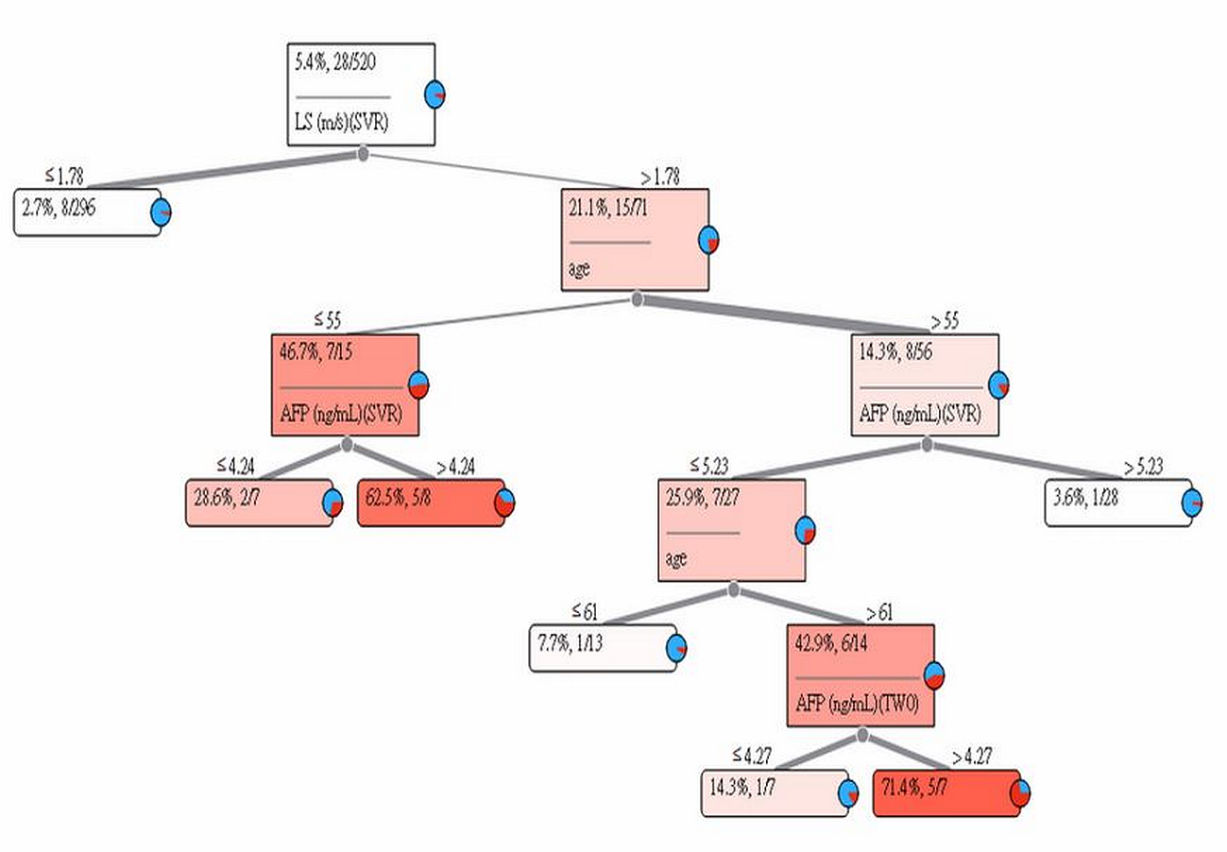


**FIGURE S1** Single decision tree classifier preliminarily trained to assist in predictor identification. The model used the significant predictors of LREs that were identified using the Cox regression model to predict the binary occurrence (yes or no) of LREs (*n* = 28), thereby minimizing information loss. Instances with missing data were excluded from classification. The parameters of the forward pruning of the decision tree were as follows: (i) binary tree; (ii) minimum *n* of instances in leaves ≥2; (iii) no splitting of nodes smaller than 5 in *n*; (iv) a maximal tree depth limit of 100; and (v) stopping when majority reaches 95%. AFP, α-fetoprotein; LRE, liver-related event; LS, liver stiffness; SVR, sustained virologic response; TW0, treatment baseline


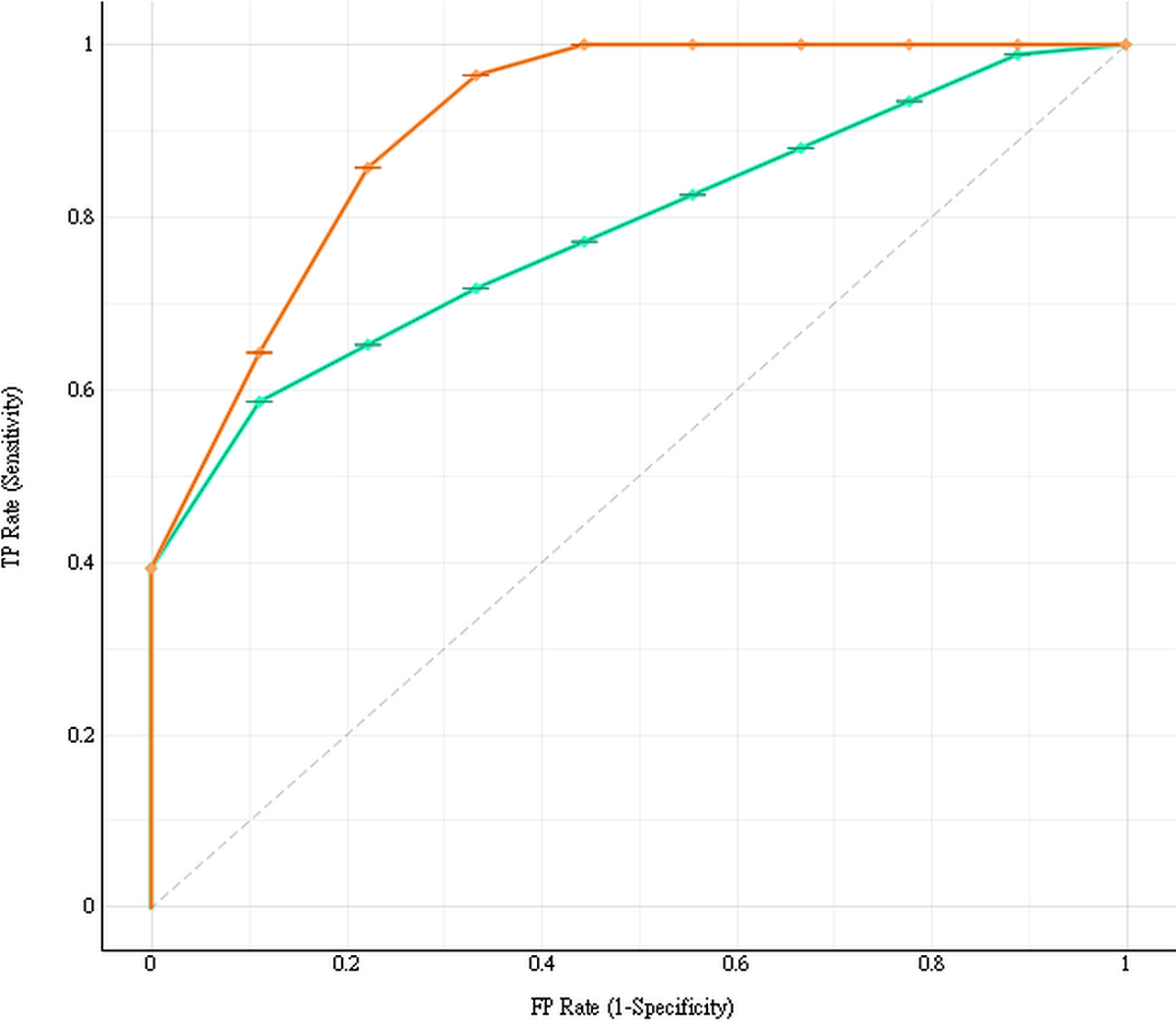


**FIGURE S2** ROC curves for LRE classification with single decision tree and ensemble random forest classifiers. Green: single decision tree; brown: random forest. Accuracy = (true positives + true negatives) / total predictions; precision = true positives / (true positives + false positives), recall = true positives / (true positives + false negatives), F1 = 2 / ([1 / precision] + [1 / recall]). AUC, area under curve; LRE, liver-related events; FP, false positive; ROC, receiver operating characteristic; TP, true positive

TABLE S1: Comparison of baseline characteristics between patients without (*n* = 457) and with (*n* = 63) clinical cirrhosis.

| Variables |  | Noncirrhosis | | Cirrhosis | |  |
| --- | --- | --- | --- | --- | --- | --- |
|  |  | Total *n* | Median (IQR)  *n*/*n* | Total *n* | Median (IQR)  *n*/*n* | *P* value |
| Age, years (TW0) |  | 457 | 55 (47–62) | 63 | 64 (57–73) | <0.001 |
| Sex, male/female |  | 457 | 227/230 | 63 | 33/30 | 0.687 |
| IFN/DAA therapy |  | 457 | 374/83 | 63 | 32/31 | <0.001 |
| AST, U/L (TW0) |  | 455 | 56 (37–87) | 62 | 85 (52–119) | <0.001 |
| ALT, U/L (TW0) |  | 457 | 74 (49–129) | 63 | 94 (54–130) | 0.173 |
| Platelets, × 10^3^/μL (TW0) |  | 457 | 168 (131–204) | 63 | 117 (95–152) | <0.001 |
| APRI (TW0) |  | 455 | 1.03 (0.63–1.98) | 62 | 2.23 (0.98–3.32) | <0.001 |
| FIB-4 (TW0) |  | 455 | 2.19 (1.34–3.49) | 62 | 4.97 (2.91–6.42) | <0.001 |
| LS, m/s (TW0) |  | 342 | 1.42 (1.20–1.93) | 45 | 2.57 (2.13–2.98) | <0.001 |

Data are presented as median (interquartile range) or *n/n*. ALT, alanine aminotransferase; APRI, aspartate-aminotransferase-to-platelet ratio index; AST, aspartate aminotransferase; DAA, direct-acting antiviral; FIB-4, fibrosis-4 index; IFN, interferon; LS, liver stiffness; TW0, treatment baseline.
